# Complex Disease Dynamics and the Design of Influenza Vaccination Programs

**DOI:** 10.1371/journal.pmed.1001553

**Published:** 2013-11-19

**Authors:** Steven Riley

**Affiliations:** MRC Centre for Outbreak Analysis and Modelling, Department of Infectious Disease Epidemiology, School of Public Health, Imperial College London, London, United Kingdom

## Abstract

Reflecting on new research by Cécile Viboud and colleagues, Steven Riley describes how understanding complex influenza dynamics can aid the design of influenza programs in China.

*Please see later in the article for the Editors' Summary*

**Linked Research Article**

This Perspective discusses the following new study published in *PLOS Medicine*:

Yu H, Alonso WJ, Feng L, Tan Y, Shu Y, et al. (2013) Characterization of Regional Influenza Seasonality Patterns in China and Implications for Vaccination Strategies: Spatio-Temporal Modeling of Surveillance Data. PLoS Med 10(11): e1001552. doi:10.1371/journal.pmed.1001552

Cécile Viboud and colleagues describe epidemiological patterns of influenza incidence across China to support the design of a national vaccination program.

For influenza vaccine programs to be optimal from the point of view of the individual at risk of infection, two conditions must be met. First, the vaccine must contain antigens that are well-matched to currently circulating strains [1]. Second, the vaccine must be administered at the right time: early enough that there is sufficient time for antibodies to rise in response to the vaccination, but not so early that protection by the vaccine wanes prior to infectious challenge [2]. The rate of waning of vaccine-induced protection against influenza is particularly high for older adults, one of the groups most at-risk of severe outcomes and often a top priority for national vaccination programs. Therefore, good knowledge of likely temporal trends in the risk of influenza infection is a necessary prerequisite for the design of optimal vaccination programs.

In this week's *PLOS Medicine*, Cécile Viboud and colleagues [3] present an extensive analysis of sentinel virological surveillance of influenzas A(H3N2) and B from China with the objective of finding epidemiological patterns that support the design of the country's first national influenza vaccination program. The authors use time series of viral isolation data from a network of sentinel hospitals, finding strong evidence for key epidemiological features of the incidence of influenza subtypes. Rather than relying on syndromic definitions or excess mortality, these biologically robust outcomes identify the patterns of circulating strains with high specificity. Despite variability in both the propensity of individuals to seek treatment and the likelihood of them being tested, virological surveillance data accurately describe the timing of peak incidence, the duration of elevated incidence (the influenza season), and periods when influenza is absent (provided testing levels are high year-round).

In many temperate populations such as the United States, knowledge of epidemiological patterns of influenza incidence has facilitated the robust design of vaccination programs [4]: incidence is strongly seasonal, with a very low risk of infection during the summer. The vast majority of infections are focused in a 6–8 week period in the winter months. Therefore, vaccination programs that are expected to last ∼6 weeks are initiated ∼12 weeks prior to the expected start of the season (the beginning of October in the Northern Hemisphere and the beginning of April in the Southern Hemisphere).

At lower latitudes, patterns are far less clear [5]. Equatorial populations such as Singapore report almost constant year-round incidence of influenza-like illness [6], while some subtropical locations, such as Hong Kong, exhibit weak biennial cycles, with their seasonality characterized primarily by a clear off-season [7]. A study of influenza patterns in Brazil, a country with a large population spanning a wide range of latitudes, revealed wave-like dynamics originating in the less populated equatorial region and travelling out towards larger temperate populations (based on excess pneumonia and influenza mortality) [8].

In their study, Viboud and colleagues were able to separate China into three epidemiological zones for influenza A(H3N2). In the temperate north, incidence peaked sharply during January and February, while in the tropical south, a longer epidemic with a lower peak was observed during April and May. The regions in the middle latitudinal zone exhibited biannual cycles with smaller incidence peaks temporally aligned with their northern and the southern neighbors.

Intriguingly, there were clear differences in the spatial patterns of influenza B compared with those of influenza A. There was little evidence of biannual cycles for influenza B, with the timing of the single peak each year closely correlated with latitude: epidemics occurred first in the north and then progressed steadily to the south. Perhaps most striking, the authors also found that the proportion of samples positive for influenza B increased from less than 20% in the northernmost provinces to almost 50% in the southernmost provinces. These observations point to fundamentally different circulation patterns between influenzas A(H3N2) and B and should motivate systematic phylogeographical and serotype studies of influenza B at the national scale in China.

The observed differences in circulation patterns between influenzas A(H3N2) and B present challenges for the design of vaccination programs at middle and lower latitudes in China. As the authors observe, the timing of peaks in the southernmost provinces is only marginally ahead of Southern Hemisphere populations and suggests that those provinces may wish to follow the Southern Hemisphere timetable. However, such a decision might be slightly premature: genetic data from even a small subset of the viral isolates used for this study could give a definitive picture of the ancestral relationship between viruses circulating in southern China relative to viruses in northern China and Southern Hemisphere populations.

A lasting legacy of the 2009 pandemic is increased interest in novel methods of manufacture for influenza vaccines [9]. Although the vast majority of vaccines delivered today arise from egg-based production systems (not substantially different from those used for the first vaccine trials ∼70 years ago), there are a number of alternative production processes under investigation that may reduce both costs and timelines [10],[11],[12]. When these technologies are fully developed, they could greatly facilitate the redesign of vaccination programs for both seasonal and pandemic influenza. As epidemiological and phylogenetic studies reveal more about the circulation of specific influenza virus subtypes in different regions of the world, it seems likely that the current system of selecting only two official vaccine strain sets per year will be refined. The results presented by Viboud and colleagues [3] suggest that rapidly expanding vaccination programs in populous mid-latitude provinces of China may provide an ideal setting in which to investigate the possible benefits of rapid vaccine production and locally-informed strain selection.
